# Wandering spleen in a postpartum female: a case report

**DOI:** 10.1097/MS9.0000000000001960

**Published:** 2024-03-18

**Authors:** Omeed Rasheed, Ali Wassouf, Aiman Abo Al Shamat, Raghad Daoud, Duaa Knaj, Ghanem Ahmad

**Affiliations:** a Department of General Surgery; b Department of Internal Medicine; c Professor of Vascular Surgery, Tishreen University, Faculty of Medicine; d Department of Oncology Medicine; e PhD student, Faculty of Medicine, Tishreen University, Lattakia; f Faculty of Medicine, Al Andalus University for Medical Sciences, Qadmus, Tartus, Syrian Arab Republic

**Keywords:** ectopic spleen, postpartum, splenopexy, splenectomy, wandering spleen

## Abstract

**Introduction and importance:**

Wandering spleen (WS) is a rare condition that occurs when the spleen is not in its normal anatomical location, but in the abdominal or pelvic cavity. The mechanism of this condition may be due to dysfunction of ligaments that fixate the spleen in its position. Female hormonal alterations during pregnancy and other unknown causes in children may also play role in an ectopic spleen.

**Case presentation:**

The authors report a case of a 34-year-old woman who presented to the emergency department with intermittent abdominal pain that persisted after childbirth without other symptoms.

**Clinical discussion:**

Clinically the symptoms are varied and abdominal pain is the most common presentation. Radiological investigation of WS include ultrasound, MRI, and CT, which is the most preferred tool. Treatments after the diagnosis include splenectomy or splenopexy either through laparoscopy or laparotomy.

**Conclusion:**

Physicians should include ectopic spleen as a differential diagnosis in a multiparous woman with the presentation of acute or chronic abdominal pain.

## Introduction

Highlights

- Wandering spleen (WS) is an uncommon medical condition characterized by weakening or loosening of the ligaments that fix that spleen in its anatomical place in the Left Upper Quadrant (LUQ).
- WS is a challenging diagnosis of exclusion because of the lack of specific symptoms, which can be asymptomatic abdominal mass or a mass associated with pain and it could not be detected unless there is torsion presenting as acute abdominal pain.
- Clinicals need to keep in mind that most cases can occurs in children and women in 20–40 years old.

Wandering spleen (WS) is a rare clinical entity condition where the spleen is found in the lower part of the abdominal or pelvic cavity instead of the normal location, which is in the Left Upper quadrant (LUQ)^1–3^. It occurs due to laxity, weakening, or absence of one or all of the peritoneal ligaments: gastrosplenic, splenorenal, or splenocyte, which holds the spleen in the hypochondrium^2,4^. The disease has no genetic background^1^. Ligament laxity can happen due to congenital factors including abnormal development or absence of three ligaments^3,4^, or acquired by a condition such as splenomegaly or pregnancy^5^. However, it mainly affects children in one third of cases, but commonly occurs in multiparous females of reproductive age in the third decade of life between 20 and 40 years^6–8^. The patient may present with acute or chronic abdominal pain. To define the WS we performed a computed tomography (CT) with contrast, which is the most optimal imaging tool for confirming the diagnosis^3,9^, also we can use alternative ways like ultrasound (US) and MRI. Due to the lack of specific symptoms, diagnosis is difficult and often incidental, unless there is torsion, presenting as acute abdominal pain^4^. This case report is reported according to Surgical CAse REport (SCARE) guideline^10^.

### Case presentation

**Figure 1:**
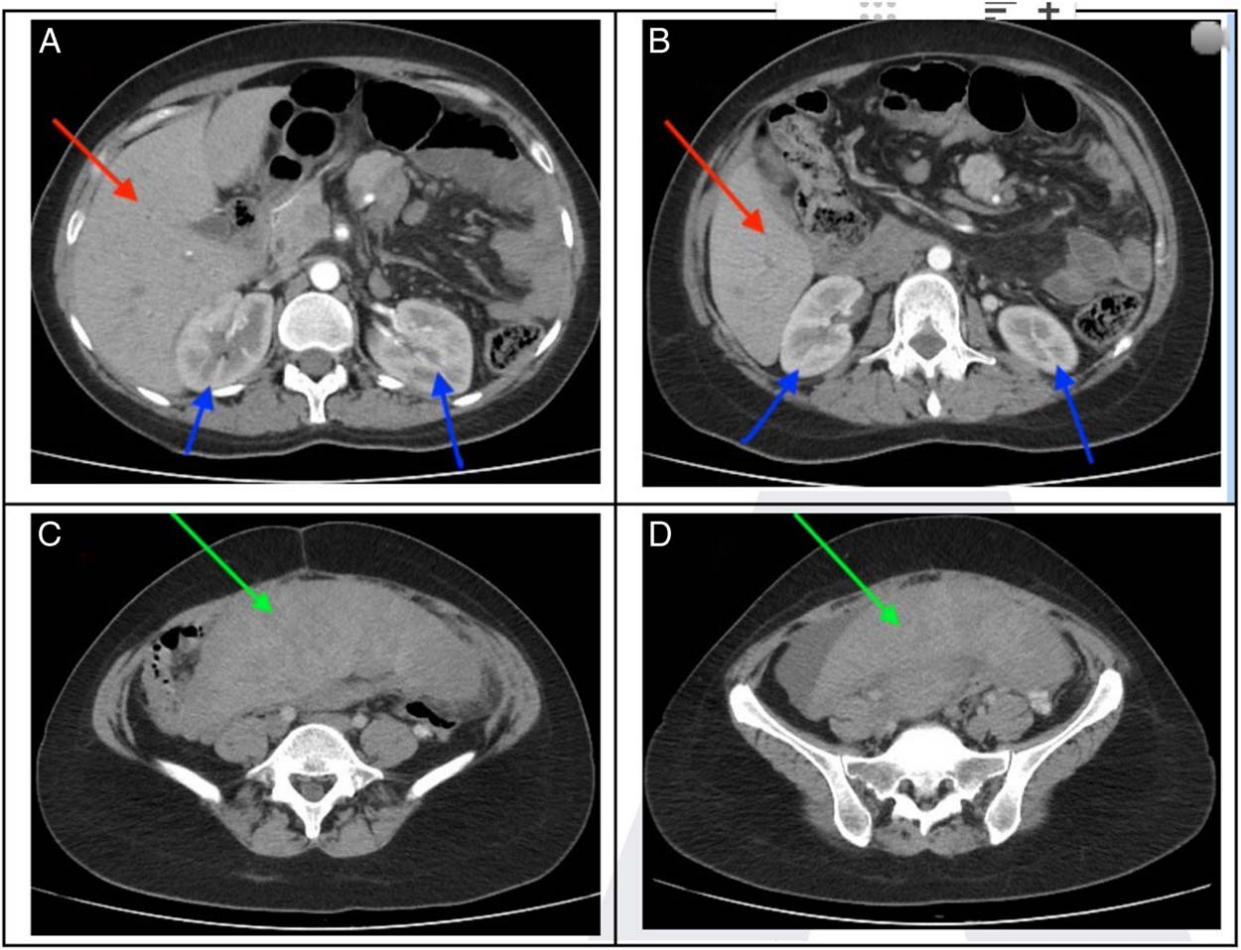
The axial CT image from A to D reveals that the spleen (green arrow) is not located in its usual position. It is situated at a lower level of the kidneys (blue arrow) and in the pelvic cavity. Liver in red arrow.

**Figure 2:**
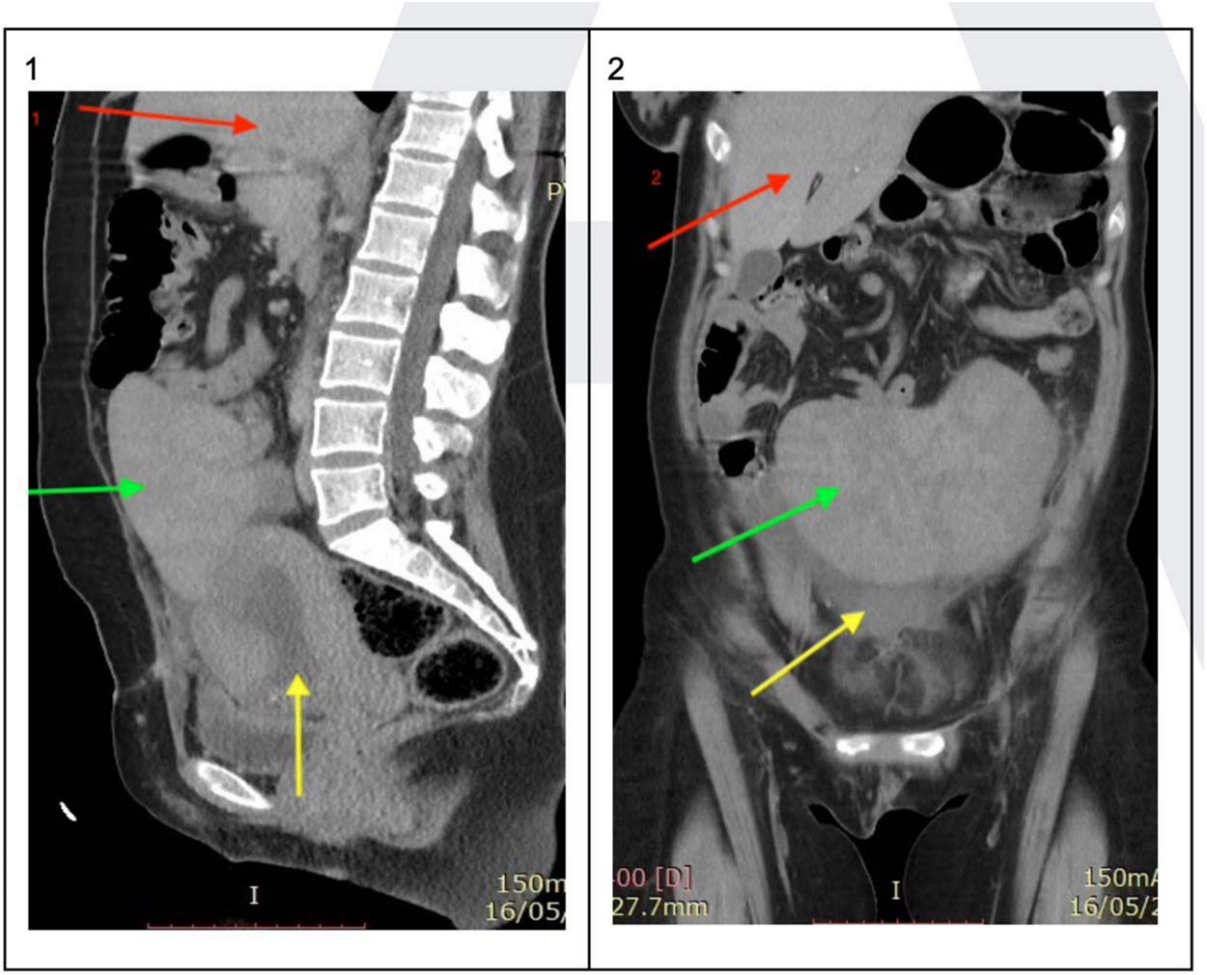
The sagittal (1) and frontal axis (2) in this women showed the spleen in pelvic cavity (green arrow) and front of uterus (yellow arrow). Liver in red arrow.

**Figure 3:**
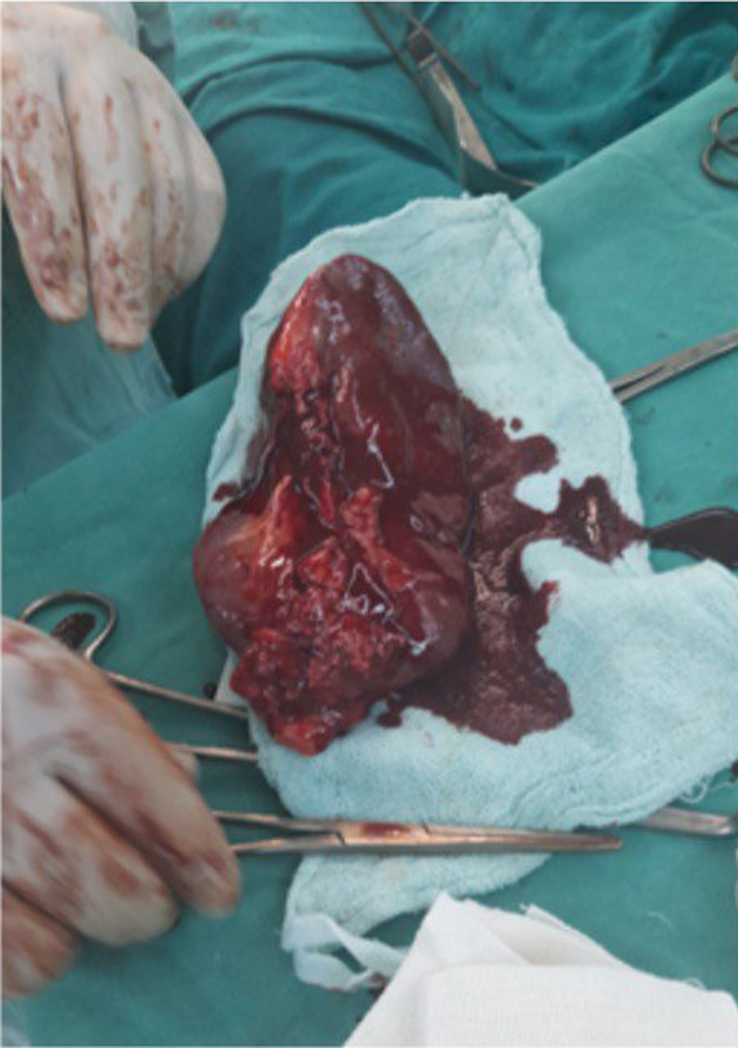
Surgery of wandering spleen in a 34-year-old women. Splenectomy was done rather than splenopexy because she had a medical history of splenic vein thrombosis.

**Figure 4:**
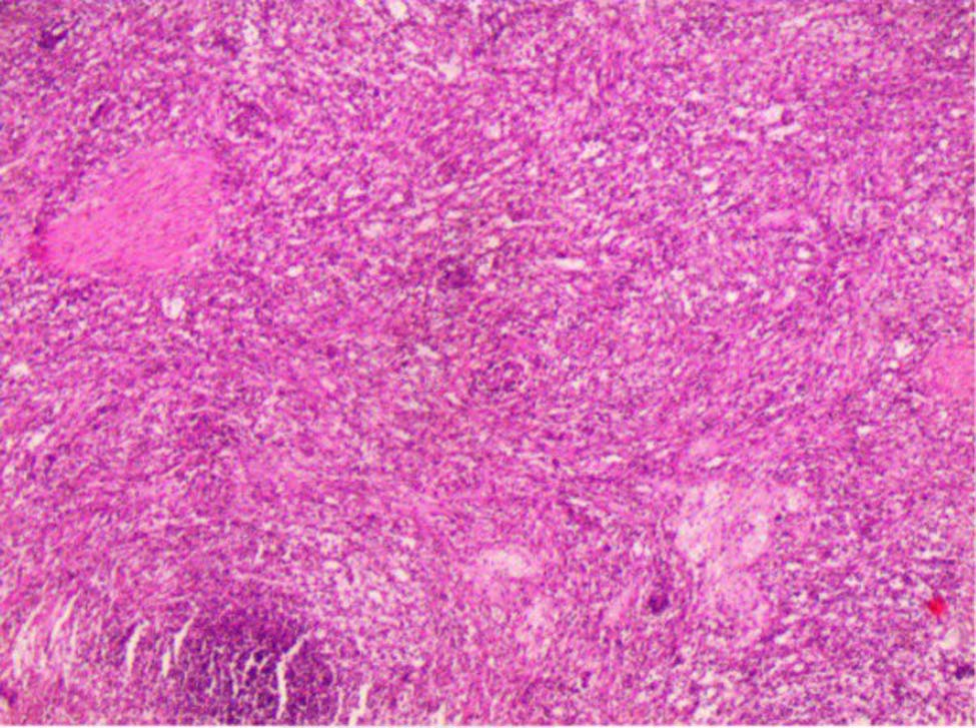
Histological study of the splenic tissue after splenectomy shows normal spleen cells and no abnormal cells.

A 34-year-old woman came to the emergency department with a complaint of persistent abdominal pain, constipation, and flatulence. Her pain began to develop during the third trimester of pregnancy and continued after cesarean birth. After childbirth, she still complained of a distended abdomen and intermittent pain. Her medical history was splenic vein thrombosis, which was treated by blood anticoagulants, with no hypertension or diabetes mellitus. At examination the patient was found to be severely distressed and in pain. upon the inspection of the abdomen, it was found to be distended and asymmetrical. The exam was otherwise normal. laboratory tests (CBC, urea, and creatinine) were ordered and all were within the normal range. An ultrasound scan was ordered and it revealed solid mass characteristics, well-defined borders, and a homogeneous splenic type similar to the spleen located in the pelvis under the kidneys. A CT scan was done and it confirmed the diagnosis of ectopic spleen, which was located in the pelvis from the midline to the left iliac fossa and no spleen in the hypochondrium (Figs 1, 2). Next, she was admitted to a general surgery department and had a splenectomy surgery (Fig. 3). The spleen was without any torsion and a biopsy of pathological examination of the sent specimens showed normal splenic tissue without any malignancy (Fig. 4). The patient’s constipation and abdominal pain ceased after surgery with good follow.

## Discussion

WS, which was first described by Van Horne in 1667^1,6^, is a rare condition that affects less than 0.2% worldwide^4,11^ and can travel from a normal location to anywhere in the pelvic or abdominal cavity^1,7^. The spleen is normally fixated in the LUQ by three ligaments: gastrosplenic, splenorenal, and splenocolic^2,5^. The ectopic spleen is most frequently diagnosed in children between 3 months and 10 years of age, women 20–40 years of age, and during pregnancy^12,13^.

Ectopic spleen, which is also called displaced spleen, floating spleen, pelvic spleen, ptotic spleen, or splenoptosis, may occur by either congenital or acquired cause^4,9,11^. The etiology of acquired causes is the laxity of the splenic ligaments due to connective tissue disorders, hormonal changes in multiparity women, splenomegaly, trauma, and previous abdominal surgery^8,12,14^. The congenital causes are due to the underdevelopment of dorsal mesogastrium leading to the absence or abnormal development of one or more of the splenic ligaments, which is associated with the elongation of the vascular pedicle^9,11^.

The clinical manifestations are different from one case to another depending on torsion or detorsion of the spleen pedicle^12^. Symptomatic patients present with acute, chronic, or mild abdominal pain and distention, vomiting, constipation, peritoneal irritation, leukocytosis, and fever^4,6^. An asymptomatic patient can be incidentally detected by US or CT as a mobile abdominal or pelvic mass^4,9^. In our case, the patient had complained of a history of chronic relapsing-remitting pain in the abdomen during the last pregnancy and long after childbirth. Differential diagnosis in this case can involve postcesarian adhesions, cecocolic torsion, or an internal abdominal hernia. Though, imaging is typically conclusive.

Multiple radiological investigations may be used to confirm the diagnosis including: US, doppler sonography, CT, or MRI, except for pregnant women, where limited radiographic procedures are safer^1,12,13^. We prefer in this condition ultrasonic B scanning, which appears to be the safer method of choice for pregnancy^13^. US technique shows the spleen does not exist in its normal location LUQ^12^. CT is accurate in diagnosing ectopic spleen and its potential complications in misdiagnosed cases^3^. However, the MRI is not a primary technique but a highly specific method^2^.

There are two methods for treatment: either laparoscopically or through laparotomy^3,9^. Splenectomy or splenopexy are the options for treatment depending on the condition of the ectopic spleen^1,3,4^. We perform splenectomy when there is torsion of the pedicle, enlargement, infarction, necrosis, rupture of the spleen, signs of hypersplenism, or splenic vein thrombosis present^2,4^. In our case, a splenectomy was preferred because the patient had a history of splenic vein thrombosis, which may have led to chronic intermittent torsion of the spleen^3,11^. Additionally, splenomegaly was an additional factor that rendered splenopexy difficult. Splenopexy is preferred when the displaced spleen is not necrotic or infracted, is in its normal size, and has no features of hypersplenism^1^. Splenopexy is better than splenectomy because it has less severe complications, especially in children. However, in asymptomatic patients, there is a 65% chance of developing complications in the next few years, so surgical treatments are favored^1–3^.

Because of its rarity and its vague symptoms, radiologists can easily misdiagnose this condition so clinicians should pay much attention to it. The early diagnosis that leads to splenopexy in childhood prevents complications. We should prescribe antibiotic prophylaxis and vaccination against pneumococcus, meningococcus, and haemophilus influenzae^14^.

## Conclusion

Herein, we presented a rare case of WS that manifested clinically as abdominal pain and constipation. Physicians should consider acute abdominal pain as a possible WS, especially in cases where the spleen is not located in its anatomical location. If the diagnosis was confirmed, surgery should be performed to relieve the symptoms.

## Ethical approval

Given the nature of the article, a case report, no ethical approval was required.

## Patient consent

Written informed consent was obtained from the patient for publication of this case report and accompanying images. A copy of the written consent is available for review by the Editor-in-Chief of this journal on request.

## Sources of funding

Not applicable.

## Author contribution

O.R., A.W., R.D., D.K., and A.A.A.S.: writing–original draft and review and editing; G.A.: supervision, review, and editing, performed patient’s imaging. All authors read and approved the final manuscript.

## Conflicts of interest disclosure

The authors declare no conflicts of interest.

## Research registration unique identifying number (UIN)

Not applicable.

## Guarantor

Ghanem Ahmad MD, Phd.

## Data availability statement

Not applicable.

## Provenance and peer review

Not commissioned, externally peer-reviewed.

## References

[1] Masroor M Sarwari MA. Torsion of the wandering spleen as an abdominal emergency: a case report. BMC Surg 2021;21:1–5. 34107944 10.1186/s12893-021-01289-x PMC8190838

[2] Parada Blázquez MJ Rodríguez Vargas D García Ferrer M. Torsion of wandering spleen: radiological findings. Emerg Radiol 2020;27:555–560. 32424633 10.1007/s10140-020-01786-1

[3] Wong A Fung KFK Wong WC. Multimodality imaging of developmental splenic anomalies: tips and pitfalls. Clin Radiol 2022;77:319–325. 35000764 10.1016/j.crad.2021.12.014

[4] Jude NN Onochie NC. Torsion of a wandering spleen. A rare cause of acute abdomen. Saudi Med J 2015;36:1490–1492. 26620993 10.15537/smj.2015.12.12363 PMC4707407

[5] Zhang P Dyer RB Holbert BL. A “wandering spleen”. Abdom Radiol (NY) 2018;43:2525–2526. 29450603 10.1007/s00261-018-1488-1

[6] Göksu M Baykan AH. Torsion of wandering spleen: a case report. J Emerg Med 2020;58:e189–e192. 32205002 10.1016/j.jemermed.2020.01.012

[7] Bhanumathi V Balkishan B Masood SV. Torsion of wandering spleen in a woman presenting as emergency. Indian J Surg 2013;75:59–61. 10.1007/s12262-012-0433-8 PMC3585543 24426389

[8] Ghazeeri G Nassar AH Taher AT. The wanderer At 12 weeks’ gestation, the patient presented with abdominal pain and a palpable mass. Am J Obstet Gynecol 2010;202:662.e1. 10.1016/j.ajog.2010.02.013 20362957

[9] Koliakos E Papazarkadas X Sleiman MJ. Wandering spleen volvulus: a case report and literature review of this diagnostic challenge. Am J Case Rep 2020;21:e925301. 32868755 10.12659/AJCR.925301 PMC7483514

[10] Sohrabi C Mathew G Maria N. The SCARE 2023 guideline: updating consensus Surgical CAse REport (SCARE) guidelines. Int J Surg Lond Engl 2023, 109:1136. 10.1097/JS9.0000000000000373 PMC10389401 37013953

[11] Anyfantakis D Kastanakis M Katsougris N. Acute torsion of a wandering spleen in a post-partum female: a case report. Int J Surg Case Rep 2013;4:675–677. 23792478 10.1016/j.ijscr.2013.05.002 PMC3710904

[12] Chauhan NS Kumar S. Torsion of a wandering spleen presenting as acute abdomen. Pol J Radiol 2016;81:110–113. 27057261 10.12659/PJR.895972 PMC4795092

[13] Clifford wj Ilano A. Accessory wandering spleen causing intestinal obstruction in pregnancy. a case report. J Int Coll Surg 1965;43:26–28. 14210827

[14] Mohseni M Kruse BT Graham C. Splenic torsion: a rare cause of abdominal pain. BMJ Case Rep 2018;2018:bcr2018224952. 10.1136/bcr-2018-224952 PMC6058114 30021736

